# Mechanical chest compression devices in the helicopter emergency medical service in Switzerland

**DOI:** 10.1186/s13049-020-00758-1

**Published:** 2020-07-25

**Authors:** Urs Pietsch, David Reiser, Volker Wenzel, Jürgen Knapp, Mario Tissi, Lorenz Theiler, Simon Rauch, Lorenz Meuli, Roland Albrecht

**Affiliations:** 1 Department of Anaesthesiology and Intensive Care Medicine, Cantonal Hospital St. Gallen, Rorschacher Strasse 95, 9007 St. Gallen, Switzerland; 2 Air Zermatt, Emergency Medical Service, Zermatt, Switzerland; 3 Department of Anaesthesiology and Pain Medicine, Inselspital, Bern University Hospital, University of Bern, Bern, Switzerland; 4 Department of Anaesthesiology and Intensive Care Medicine, Friedrichshafen Regional Hospital, Röntgenstraße 2, 88048 Friedrichshafen, Germany; 5 Swiss Air-Ambulance, Rega (Rettungsflugwacht / Guarde Aérienne), Swiss Air-Rescue, Zurich, Switzerland; 6 Institute of Mountain Emergency Medicine, Eurac Research, Bozen, Italy; 7 Department of Anaesthesiology and Intensive Care Medicine, F. Tappeiner Hospital, Merano, Italy; 8 Department of Vascular Surgery, St. Gallen Cantonal Hospital, St. Gallen, Switzerland

**Keywords:** AutoPulse, Mechanical chest compression devices, Cardiopulmonary arrest, Cardiopulmonary resuscitation, Helicopter emergency medical services, Load-distributing band CPR device

## Abstract

**Background:**

Over the past years, several emergency medical service providers have introduced mechanical chest compression devices (MCDs) in their protocols for cardiopulmonary resuscitation (CPR). Especially in helicopter emergency medical systems (HEMS), which have limitations regarding loading weight and space and typically operate in rural and remote areas, whether MCDs have benefits for patients is still unknown. The aim of this study was to evaluate the use of MCDs in a large Swiss HEMS system.

**Materials and methods:**

We conducted a retrospective observational study of all HEMS missions of Swiss Air rescue Rega between January 2014 and June 2016 with the use of an MCD (Autopulse®). Details of MCD use and patient outcome are reported from the medical operation journals and the hospitals’ discharge letters.

**Results:**

MCDs were used in 626 HEMS missions, and 590 patients (94%) could be included. 478 (81%) were primary missions and 112 (19%) were interhospital transfers. Forty-nine of the patients in primary missions were loaded under ongoing CPR with MCDs. Of the patients loaded after return of spontaneous circulation (ROSC), 20 (7%) experienced a second CA during the flight. In interhospital transfers, 102 (91%) only needed standby use of the MCD. Five (5%) patients were loaded into the helicopter with ongoing CPR. Five (5%) patients went into CA during flight and the MCD had to be activated. A shockable cardiac arrhythmia was the only factor significantly associated with better survival in resuscitation missions using MCD (OR 0.176, 95% confidence interval 0.084 to 0.372, *p* < 0.001).

**Conclusion:**

We conclude that equipping HEMS with MCDs may be beneficial, with non-trauma patients potentially benefitting more than trauma patients.

## Introduction

High-quality chest compressions, minimal hands-off times, and early external defibrillation are crucial for survival with good neurological outcome in patients with cardiac arrest (CA), as underlined by the current resuscitation guidelines [1–3]. However, maintaining high-quality cardiopulmonary resuscitation (CPR) is often challenging in the prehospital setting, in particular during evacuation and transport [4, 5]. Over the past years, a number of institutions providing emergency medical care have introduced mechanical chest compression devices (MCDs) in their CPR protocols [6–8]. AutoPulse® (Zoll Medical, Chelmsford, MA, USA) and LUCAS™ (Physio-Control, Redmond, WA, USA) are the two most frequently used devices worldwide [9].

The Circulation Improving Resuscitation Care (CIRC), LUCAS in cardiac arrest (LINC) and Prehospital Randomized Assessment of a Mechanical Compression Device (PARAMEDIC) trials have assessed the effect of MCDs on survival. In these large prehospital trials, no benefit in patient outcome was shown, hence the routine use of MCDs is not recommended in ERC and AHA CPR guidelines [10–12]. These CPR guidelines, however, suggest the use of MCDs in situations where providing manual chest compressions is impractical, as a bridge to advanced therapies, or when provider safety is compromised. This applies, for example, to helicopter emergency medical services (HEMS). Common European EMS helicopters such as the H145, EC135 or AW109 have limited space and height in the cabin, and providing high-quality chest compressions in a flying helicopter or in a remote environment with limited personnel and space is often impossible.

In Switzerland, four organizations provide physician-staffed HEMS operations 24/7, including primary (pre-hospital retrieval) and secondary (inter-hospital transfer) missions around the clock.

The less space available for the HEMS crew and patient during the transport, the greater the advantages of MCDs. Many medical professionals therefore support equipping HEMS with MCDs. However, this could result in frequent transport of patients in persistent CA with dismal prognoses, thereby not only producing direct costs for transport and treatment but also harming other patients indirectly by blocking valuable emergency medical care resources [13].

In this retrospective study, we sought to investigate frequency and circumstances surrounding use of an MCD in a large Swiss helicopter emergency medical service (HEMS), and to analyze use in terms of compliance with CPR guidelines and patient outcome.

## Methods

### Design

We conducted a retrospective study examining the period lasting from December 31, 2013, to June 30, 2016 (912 days). The institutional review board of the Bern cantonal ethics committee (KEK) reviewed the study design and granted permission for use of patient data without individual consent, according to the federal act on research involving human beings, and the ordinance on human research with the exception of clinical trials. The permission covered the use of patient data collected during the HEMS operation and related hospital stays (KEK Bern, September 13, 2016, reference number 2016–01473).

### Setting and population

Swiss Air-Rescue (Rega) conducts around 11,000 HEMS missions annually. In 2013 Rega operated 13 helicopter bases, which are distributed throughout the country in such a way that they can fly to any location at any time within its operational area within 15 min. The helicopter fleet comprises 7 Eurocopter EC145 machines, stationed at the lowland bases in Zurich, Basel, Bern, Lausanne and St. Gallen, and 11 AgustaWestland Da Vinci located at the mountain bases in Untervaz, Locarno, Erstfeld, Samedan, Wilderswil, Mollis and Zweisimmen. All 13 helicopters are equipped for winch operations and night-time flying.

In Switzerland, the HEMS crew includes a pilot, a paramedic and an emergency physician. The paramedic also serves as winch operator and is therefore not available to treat the patient on-site in case of a winch rescue. In 2013, the AutoPulse® device was added to the standard equipment of the Rega HEMS. Primary missions take place in a large variety of environments and situations, ranging from providing support for ground ambulances in urban areas to autonomous operations in extreme mountainous terrain. We included every operation (primary and secondary mission) during the investigation period.

### Protocol

After every HEMS operation, a variety of information is recorded in the Rega operations database (SAP Software), including whether the MCD was used or not. In a first step, we filtered the database for all operations where the use of the MCD had been reported, and we excluded other operations. For the included operations, we then gathered the physicians’ medical operations report forms from the Rega archive. In a second step, we examined these reports and entered key information in a database (Labkey). For the remaining operations, we identified the ones in which the patient was transported to a hospital, and we asked the respective hospitals to provide us with the discharge letters of these patients. In a further step, we analyzed the hospitals’ discharge letters and also integrated their key information into our database. The fourth and final step was to analyze the collected data.

### Statistics

The characteristics of all patients were compared using descriptive statistics.

To further elaborate on factors that are associated with an impaired outcome during AutoPulse® resuscitations, univariate and multivariate logistic regression models were built including the binary variables sex, CPR by layperson, shockable cardiac rhythm as well as the continuous variables age and number of shocks applied. All variables with *p* < 0.1 in univariate analysis were included in the multivariate analysis.

The binary variable diagnosis (trauma versus medical diagnosis) was excluded due to a separation problem (i.e. only one survivor in the trauma group). To further assess the impact of the diagnosis variable, a Firth’s bias reduced logistic regression model was performed to evade separation [14]. The resulting estimate and standard error for the diagnosis variable remained very large. Hence, the diagnosis variable was excluded for the analysis to avoid bias.

Due to the relevant number of missing data in the variables CPR by layperson (*n* = 70), shockable rhythm (*n* = 62), and survival (*n* = 70), a sensitivity analysis using multiple imputations was performed*.* All variables of the model were used for imputation in the target variable using the pmm method, generating 10 imputed data sets. Multiple imputation was performed using the *mice* R-Package [15].

Two-sided *p*-values of < 0.05 were considered statistically significant. All statistical analyses were performed using R Studio 3.6.0 on macOS 10.15.4.

## Results

**Fig. 1:**
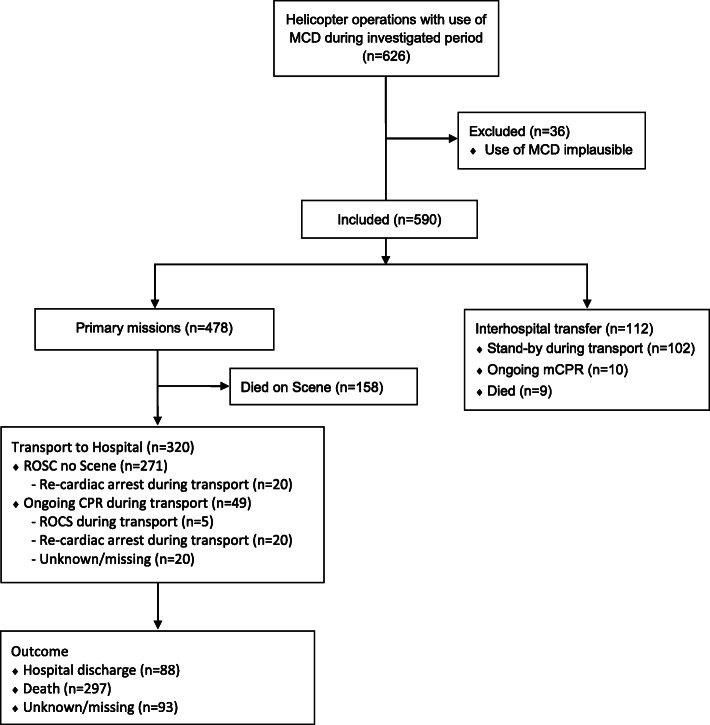
Flow Diagram of Scene Calls of Primary and Secondary Missions

In the study period, Rega conducted a total of 22,365 HEMS operations. In 626 operations (3%) the use of the MCD was reported: 590 of those operations (2.6%) could be included (Fig. 1). We had to exclude 36 scene calls in which we could not discern why the MCD was used. One part of this excluded group consisted of calls to the scene where the medical report described a scenario that was highly unlikely to have required the use of a MCD. In these cases, we suspected errors were made in entering information into the database. The other part consisted of operations where the medical report was missing, destroyed, or unreadable. The included cases could be divided into 478 (81%) primary and 112 (19%) secondary missions. 74% of the analysed patients were male, and the mean age was 59 ± 17 years. Patient and mission characteristics of primary and secondary missions are described in Tables 1 and 2.

**Table 1:** Clinical Characteristics of Primary HEMS Missions

Variable	Women, ***n*** = 101 (21%)		Men, ***n*** = 377 (79%)	
	Trauma ***n*** = 17 (17%)	Non-Trauma ***n*** = 84 (83%)	Trauma ***n*** = 53 (14%)	Non-Trauma ***n*** = 324 (86%)
Age, mean ± SD	59 ± 17	58 ± 17	53 ± 17	58 ± 17
Autopulse standby	–	–	–	15 (5%)
Asphyxiation/Drowning	9 (53%)	–	11 (21%)	–
Avalanche/Hypothermia	2 (12%)	–	8 (15%)	–
Blunt multiple trauma	6 (35%)	–	32 (60%)	–
Other/Unknown	–	–	2 (4%)	–
Presumed cardiac	–	27 (32%)	–	144 (44%)
Respiratory	–	–	–	4 (1%)
Other/Unknown	–	57 (68%)	–	176 (54%)
Defibrillation	–	40 (48%)	5 (9%)	198 (61%)
In flight	–	1 (1%)	–	12 (4%)
AED	–	3 (4%)	–	17 (5%)
Initial cardiac rhythm				
VF/VT	1 (6%)	33 (39%)	1 (2%)	168 (52%)
PEA/Asystole	16 (94%)	33 (39%)	49 (92%)	109 (34%)
Unknown	–	18 (21%)	3 (6%)	47 (15%)
Lay CPR before EMS arrival	7 (41%)	36 (43%)	25 (47%)	187 (58%)
ROSC	4 (24%)	59 (70%)	20 (38%)	225 (69%)
Intubation	17 (100%)	84 (100%)	53 (100%)	309 (95%)
Transport with ongoing CPR	3 (17%)^*^	8 (10%)	12 (23%)^*^	26 (8%)
Time on scene				
< 30 min	1 (6%)	13 (15%)	10 (19%)	90 (28%)
30–60 min	–	19 (23%)	23 (43%)	78 (24%)
> 60 min	–	4 (5%)	–	4 (1%)
Unknown	–	–	–	–
Outcome				
Hospital discharge	–	11 (13%)	1 (2%)	76 (23%)
Death	17 (100%)	48 (57%)	52 (98%)	180 (56%)
Unknown	–	25 (30%)	–	68 (21%)

Percentages are calculated within the columns

*SD* Standard Deviation, *AED* Automated External Defibrillator, *VF* Ventricular Fibrillation, *VT* Ventricular Tachycardia, *PEA* Pulseless Electrical Activity, *CPR* Cardiopulmonary Resuscitation, *EMS* Emergency Medical Services, *ROSC* Return of Spontaneous Circulation

**Table 2:** Clinical Characteristics of Secondary HEMS Missions

Variable	Overall ***N*** = 112	Women ***n*** = 29 (26%)	Men ***n*** = 83 (74%)
Age, mean ± SD	65 ± 15	64 ± 15	65 ± 14
Presumed Cardiac	54 (48%)	23 (79%)	31 (37%)
Respiratory	5 (5%)	–	5 (6%)
Other / Unknown	53 (47%)	6 (21%)	47 (57%)
Autopulse mode during transport			
Stand-by	102 (91%)	25 (86%)	76 (92%)
Active (ongoing CPR)	9 (8%)	4 (14%)	6 (7%)
Stand-by/active	1 (1%)	–	1 (1%)
Outcome			
Hospital discharge	49 (44%)	12 (41%)	37 (45%)
Death	31 (28%)^*^	10 (35%)	21 (25%)
Unknown	32 (29%)	7 (24%)	25 (30%)

Percentages are calculated within the columns

*CPR* Cardiopulmonary Resuscitation

*N*=112, *Autopulse active/ongoing mCPR =100% death

### Primary missions

In 158 cases (33%), the death of the patient was documented on the scene. In the remaining 320 missions (67%), the patient was transported to a hospital. The transported patients could be divided into a first group of 49 cases (15%) where CPR was ongoing when the patient was loaded into the HEMS and a second group of 271 cases (85%) where the patient had regained spontaneous circulation before being loaded into the HEMS (Fig. 1). In the first group, ROSC could be achieved during transport in five patients (10%). However, only one patient (2%) survival to hospital discharge; 46 patients (94%) died in the hospital; In two cases (4%) the endpoint remains unknown because of missing hospital reports. Of the patients in the second group, 20 (7%) experienced a second CA during the flight, two (1%) could be resuscitated with a single defibrillation attempt, and the remaining 18 (7%) needed full CPR efforts, including the use of the MCD. Those 18 patients died in hospital. The remaining 251 patients (93%) from this group with ROSC on the scene remained haemodynamically stable during the flight.

In two cases (0.4% of all primary operations) a malfunction of the MCD was documented. One malfunction was due to rough terrain hindering the correct positioning of the device; the second was an unexplained inability of the device to start chest compressions when cardiac arrest occurred in flight.

The logistic regression models show that presenting with a shockable cardiac arrhythmia is the only factor significantly associated with better survival in resuscitation missions using MCDs (OR 0.176, 95% confidence interval [CI] 0.084 to 0.372, *p* < 0.001). The odds of survival are more than five times greater in patients with a shockable cardiac arrhythmia compared to patients with a non-shockable cardiac rhythm. This finding was robust in the sensitivity analysis using multiple imputation methods for missing data.

**Fig. 2:**
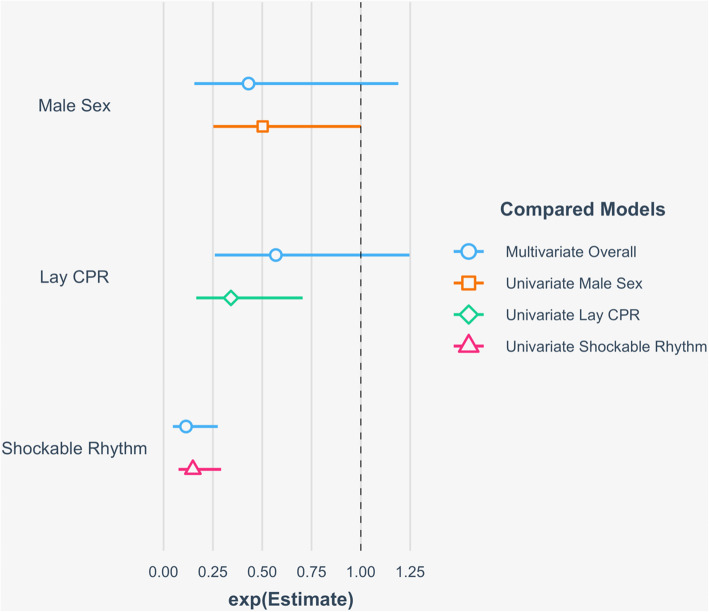
Univariate compared to Multivariate Logistic Regression Models on Survival in Resuscitations using MCD. Figure based on the Complete-Case Analysis, see Tables 4 and 5. exp. (Estimate): Representing odds ratios, 1.00 indicates no difference in survival. The line around the dot indicates the 95% Confidence interval of the odds rat

Univariate logistic regression models showed that male sex and layperson CPR were associated with better survival as well (Table 3; Fig. 2). However, these effects were no longer statistically significant when adjusted for the type of cardiac arrhythmia (shockable versus non-shockable) in the multivariate analysis (Tables 4 and 5; Fig. 2).

**Table 3:** HEMS Mission Location

Location (primary and secondary missions)	*N* = 590
Hospital (Interhospital Transfer/secondary missions)	*N* = 112 (19%)
Public place (primary missions)	*N* = 360 (61%)
Mountainous or remote locations (primary missions) (5 missions with ongoing CPR during winch rescue)	*N* = 118 (20%)

*N* = 590

**Table 4:** Multivariate Logistic Regression Models on Survival in Resuscitations using Autopulse

Variable	Multivariate Model *(Complete Case Analysis)*			Multivariate Model *(Multiple Imputation)*		
	OR	95% C.I.	*p*-value	OR	95% C.I.	*p*-value
Male Sex	0.456	0.167 to 1.248	0.126	0.672	0.338 to 1.336	0.258
CPR by Lay	0.554	0.255 to 1.202	0.135	0.610	0.307 to 1.214	0.111
Shockable Rhythm	0.176	0.084 to 0.372	< 0.001	0.092	0.073 to 0.286	< 0.001

*OR* Odds ratio, *95% C.I.* 95% Confidence interval of the odds ratio

Complete-Case Analysis: Included observations *n* = 315, 163 missing observations

Multiple-Imputation Model: No of Imputations = 10, No of Iterations = 50, Method = PMM

Female sex, not receiving lay CPR and non-shockable cardiac rhythm served as the reference groups in both models

**Table 5:** Univariate Logistic Regression Models on Survival in Resuscitations using Autopulse

Variable	Univariate Models *(Complete Case Analysis)*			Univariate Models *(Multiple Imputation)*		
	OR	95% C.I.	*p-*value	OR	95% C.I.	*p*-value
Age	0.999	0.985 to 1.013	0.840	0.999	0.985 to 1.012	0.816
Male Sex	0.502	0.252 to 1.000	0.050	0.325	0.284 to 1.012	0.057
CPR by Lay	0.341	0.165 to 0.705	0.004	0.406	0.226 to 0.729	0.004
Shockable Rhythm	0.148	0.076 to 0.291	< 0.001	0.128	0.065 to 0.255	< 0.001
No of Shocks	0.938	0.858 to 1.025	0.158	0.948	0.872 to 1.031	0.214
*Trauma*	*46.08*	*6.279 to 338.2*	*< 0.001*	*30.54*	*4.014 to 232.3*	*0.001*

*OR* Odds ratio, *95% C.I.* 95% Confidence interval of the odds ratio

Complete-Case Analysis: Included observations *n* = 315, 163 missing observations

Female sex, not receiving lay CPR and non-shockable cardiac rhythm served as the reference groups in both models

Trauma variable: Due to the low number of survivors in patients with a trauma diagnosis (*n* = 1) the regression estimates are biased (separation); estimates are presented for completeness purpose only

Multiple-Imputation Model: No of Imputations = 10, No of Iterations = 50, Method = PMM

### Secondary missions

Amongst the secondary operations, 102 (91%) did not require use of the MCD, which was preinstalled and on standby. Of the rest, five (5%) patients were loaded into the helicopter with ongoing CPR; ultimately, all of these patients died in the hospital. The remaining five (5%) patients went into cardiac arrest during the flight and CPR was initiated, with one of them achieving ROSC before landing (Fig. 1).

## Discussion

We found that in successfully resuscitated patients, the risk of cardiac arrest re-occurring during the flight was high during both primary and secondary missions. Therefore, MCDs are beneficial for CPR during airlift to the hospital. Employing MCDs was feasible and safe in the HEMS; standing orders and crew training were carefully implemented, as previously described [16]. There were only two MCD malfunctions (one on the scene, one in flight).

Unnecessary prolonged CPR before terminating efforts is reported in several studies and should be avoided, as it blocks the HEMS crew and therefore wastes EMS and hospital resources [17, 18]. In our study, the time until ROSC or termination of CPR was usually < 30 min, indicating determined decision-making on the scene in routine cases. However, in patients being airlifted with ongoing CPR, we found that there is a relevant proportion of cases without recommended indication regarding reversible causes of cardiac arrest, suggesting an individual interpretation of the situation. The poor prognosis in this group of patients, with only 3% survival at discharge, is in accordance with recent studies [19, 20]. However, precise criteria for patient selection and timing are still lacking. On the other hand, there is an increasing amount of literature showing that carefully selected patients may benefit from early transport with ongoing CPR for further treatment [19–21].

A relevant finding of our study was the fact that in 20 of 271 patients with ROSC (7%), a second cardiac arrest occurred. A pre-installed MCD was a safe and fast way to initiate chest compressions in flight. However, in many HEMS worldwide, manpower and space in the cabin are severely limited and it is impossible to install a MCD or to perform sufficient and efficient manual chest compressions in flight. Thus our findings support the need for a preinstalled device in this patient group to prevent a relevant delay in CPR in case of re-arrest.

A shockable cardiac arrhythmia was associated with a five times higher chance for survival in the multivariate logistic regression analysis (OR 0.176, 95% CI 0.084 to 0.372, *p* < 0.001). These findings are in a line with previous data which demonstrated that survival was best in the group with shockable rhythms in OHCA [22]. These findings could help create further SOPs on the indication, use and duration of MCDs.

Despite the evidence against general use of MCDs, case reports and the recent ERC and AHA guidelines report beneficial effects during prolonged CPR in special circumstances, such as accidental hypothermia, intoxication, or pulmonary embolism [23–25]. However, providing high-quality chest compressions with minimal hands-off time is challenging during evacuation, e.g., in alpine environments and during transport in a helicopter. Accidental hypothermia from a fall in a crevice, submersion in ice water, or burying in an avalanche is more common in HEMS accidents than in accidents treated by ground-based EMS [25, 26]. Therefore, according to the current CPR recommendations, a robust and feasible way to perform CPR is crucial in this patient group.

Few studies have looked at the feasibility of the different MCDs in mountain HEMS. In general, there are different underlying mechanisms and theories driving MCDs, the cardiac pump theory (LUCAS™) and the thoracic pump theory (Autopulse®) [27]. Requirements for HEMS with winch rescue are different from requirements for ground-based EMS. In a simulation study and additionally in a case series, Pietsch et al. demonstrated the feasibility of using MCDs, even in remote areas and adverse environments requiring winch rescues with ongoing chest compression [4, 28]. In our study, one of the 69 trauma patients with known data survived to hospital discharge. This may indicate that the trauma patient sample was too small, or that the wrong patients were transported to the hospital.

All of the existing MCD studies, and even the recent Cochrane review, excluded studies explicitly including patients with cardiac arrest caused by trauma, drowning, hypothermia and toxic substances. These conditions are mostly excluded from cardiac arrest intervention studies because they have a different underlying pathophysiology, therapy and thus prognosis [29]. To our knowledge there is no study reporting data on outcomes of traumatic CA and MCDs so far. Thus, this study would be the first reporting data in this field of non-cardiac-caused CAs and MCDs. In contrast, hospital discharge rates ranged between 13% in women and 23% in men suffering non-traumatic cardiac arrest, indicating that employing MCDs resulted in very good survival rates in this group. Our clear findings against the use of MCDs in traumatic CA could potentially influence resuscitation guidelines in the future.

MCDs offer additional benefits for the HEMS crew. During a simulated helicopter transport, Rehatschek et al. showed that MCDs improved the efficacy of chest compressions, reduced physical stress, and led to enhanced cognitive performance of the EMS crew as compared to manual CPR [30]. This may enable better monitoring of clinical developments, and leaves hands free for additional interventions. However, a theoretical basis and evidence regarding those positive effects on human error, as well as data on non-technical skills, are still limited.

An obvious reason to implement MCDs in HEMS is to ensure conformity with flight transport regulations, such as the requirement for the entire crew to have latched seatbelts during ongoing chest compressions in flight. Further, with a growing number of contagious diseases (e.g., COVID-19), MCDs can enhance safety while still allowing provision of high-quality CPR with maximal distance to the patient on the scene and during transport.

The strength of our study is the large patient cohort we examined. The HEMS missions in our study are very diverse, consisting of a mixture of urban, rural, mountainous, and alpine areas, thus the results may be extrapolated to other EMS settings. Due to the retrospective study design we could investigate application of MCDs in daily practice of HEMS without the artificial limitations of a prospective trial protocol. In addition, the fact that the involved HEMS were physician-staffed meant that the course of CPR efforts in our study was not heavily influenced by legal considerations, since physicians could pronounce a patient dead on the scene. In contrast, paramedic-staffed services – depending on local legislation and protocols – might require extensive CPR efforts before termination, thus altering the role of MCDs.

The retrospective observational nature of our study has several limitations as well. First, there was no systematically gathered information regarding MCDs from the HEMS missions except for the question of whether the device was used or not. We extracted all relevant information from the medical HEMS reports, but quality varied greatly. Second, there may have been missions where a MCD was used but not documented in the HEMS database. Since we were unable to check all missions manually, we could not include them, and therefore we might have underestimated the true proportion of HEMS missions involving MCDs, a potential source of selection bias. Third, a multivariable logistic regression was performed adjusting the analysis for confounding factors, however, residual confounding due to unmeasured factors is possible. And last, we could not compare our findings with a control group since identifying a set of comparable HEMS missions in the years preceding the implementation of MCDs in our organization was not possible.

## Conclusion

Equipping HEMS with MCDs may be beneficial to treat patients with non-traumatic cardiac arrests.

## Data Availability

Not applicable.

## Abbreviations

AED: Automated external defibrillator
AHA: American Heart Association
CA: Cardiac arrest
CPR: Cardiopulmonary resuscitation
ECMO: Extracorporeal membrane oxygenation
ECPR: Extracorporeal cardiopulmonary resuscitation
ERC: European Resuscitation Council
HEMS: Helicopter emergency medical service
ILCOR: International Liaison Committee on Resuscitation
KEK: Cantonal Ethics Committee
LUCAS: Lund University Cardiac Assist System
MCD: Mechanical chest compression device
NACA: National Advisory Committee for Aeronautics
OHCA: Out-of-hospital cardiac arrest
